# Integrating molecular epidemiology and social network analysis to study infectious diseases: Towards a socio-molecular era for public health

**DOI:** 10.1016/j.meegid.2016.05.042

**Published:** 2016-12

**Authors:** Tetyana I. Vasylyeva, Samuel R. Friedman, Dimitrios Paraskevis, Gkikas Magiorkinis

**Affiliations:** a Department of Zoology, University of Oxford, South Parks Road, OX1 3PS Oxford, United Kingdom; b Institute for Infectious Disease Research, National Development and Research Institutes, New York, NY 10010, USA; c Department of Hygiene, Epidemiology, and Medical Statistics, Athens University Medical School, 75, M. Asias Street, Athens 115 27, Greece

**Keywords:** Social networks, Molecular epidemiology, Phylodynamics, Phylogenetics, Infectious diseases, Viruses

## Abstract

The number of public health applications for molecular epidemiology and social network analysis has increased rapidly since the improvement in computational capacities and the development of new sequencing techniques. Currently, molecular epidemiology methods are used in a variety of settings: from infectious disease surveillance systems to the description of disease transmission pathways. The latter are of great epidemiological importance as they let us describe how a virus spreads in a community, make predictions for the further epidemic developments, and plan preventive interventions. Social network methods are used to understand how infections spread through communities and what the risk factors for this are, as well as in improved contact tracing and message-dissemination interventions. Research is needed on how to combine molecular and social network data as both include essential, but not fully sufficient information on infection transmission pathways. The main differences between the two data sources are that, firstly, social network data include uninfected individuals unlike the molecular data sampled only from infected network members. Thus, social network data include more detailed picture of a network and can improve inferences made from molecular data. Secondly, network data refer to the current state and interactions within the social network, while molecular data refer to the time points when transmissions happened, which might have happened years before the sampling date. As of today, there have been attempts to combine and compare the data obtained from the two sources. Even though there is no consensus on whether and how social and genetic data complement each other, this research might significantly improve our understanding of how viruses spread through communities.

## Highlights

• We summarise and analyse the roles of molecular evolution studies in molecular epidemiology of infectious diseases.
• We review how social network and molecular sequence data have been integrated in the past.
• We show how integrating social network and molecular evolution approaches may change the study of infectious diseases.

Molecular epidemiology of infectious diseases aims to harness molecular (nucleotide or amino acid) sequences to study the ecology and dynamics of pathogens (Foxman and Riley, 2001). With the recent advances in nucleotide sequencing (i.e. high throughput sequencing technologies) which allow faster and more affordable sequencing of pathogens (Grada and Weinbrecht, 2013), vast amounts of genetic data can be produced faster, cheaper and more efficiently than ever. This data-driven revolution has generated expectations with respect to more effective use of molecular sequences for scientific and public health purposes. Even though techniques have been developed to use these new forms and structures of data in research on spread, distribution, treatment and prevention of infectious disease epidemics (Kuhnert et al., 2011, Hartfield et al., 2014), it is still unclear what can be inferred by means of next generation sequencing (NGS, refers to multiple current sequencing techniques) data and, most importantly, how can we exploit them as much as possible.

Here we will review theoretical developments and applications of using molecular sequences to study the spread of infectious diseases and more particularly human viruses. We first use a “frequently asked questions” approach: we answer questions that we have heard during discussions with researchers not directly related to the field of molecular epidemiology. Then we focus on the emerging field of integrating social network data with molecular sequences, as we believe we are entering an exciting new era of socio-molecular epidemiology.

## 1 Epidemics on a macro scale: inferring the statistics of epidemic spread

### 1.1 Can we use molecular sequences to estimate traditional epidemiological parameters such as the basic reproductive number?

Genomic and epidemiological data can be used to estimate infectious disease spread parameters as reliably as using mathematical models or detailed epidemiological contact-tracing information. Parameters of interest usually include *R*_0_, the basic reproductive number, which can be thought of as the number of secondary infections attributed to one infected individual in a completely susceptible population, and the generation time, which is the time that it takes one infected person to transmit a virus to another person (Anderson and May, 1991). Estimating *R*_0_ is crucial for the prognosis of an epidemic and for developing strategies to stop the epidemic spread; knowing the generation time is important as it tells us when in the course of epidemic interventions can be more effective to prevent transmissions. Using genetic data *R*_0_ has been estimated for different viruses, including multiple types and subtypes of Hepatitis C (HCV) (Pybus et al., 2001, Magiorkinis et al., 2013), HIV (Magiorkinis et al., 2014), and Influenza A (Fraser et al., 2009). By assuming a range of durations of the infectivity periods and different proportions of transmitters in a population, we were able to estimate the generation time of HCV in different populations in Greece by combining genetic and surveillance data (Magiorkinis et al., 2013). During the 2009 Influenza outbreak and the 2014 Ebola outbreak researchers have shown that epidemiological parameters inferred through molecular data are similar to those coming from the count-based epidemiological studies (Fraser et al., 2009, Alizon et al., 2014), suggesting that molecular data are reliable for epidemiological parameters estimations.

### 1.2 Can we use molecular sequences to monitor/infer the spatiotemporal spread of an epidemic within a population?

Firstly, there are multiple examples where genomic data have been used to improve epidemiological surveillance. For instance, public health efforts to control influenza outbreaks were strengthened by developing a genomic surveillance system that allows monitoring the temporal trends in virus mutations and planning preventive efforts (including vaccine design) for the following years (Russell et al., 2008). Surveillance systems like that have become more affordable/available with the appearance of NGS data. Retrospectively, data on the air transportation network and influenza A surveillance were used to show that, as expected, the spread of influenza H3N2 can be explained by air passenger flows (Lemey et al., 2014). Another example of the use of phylogenetics to enhance infectious disease surveillance is an approach used to define the clustering of HCV infections. Researchers from Canada used genetic data sequentially collected from people who inject drugs (PWID) to define the intra-host genetic distance (Olmstead et al., 2015). They then classified “recent transmission clusters” if the between-hosts genetic distance fell within the intra-host distance intervals. This approach allows monitoring small viral infections outbreaks within the PWID group, which, to the extent to which it can be done in real time, can assist in transmission-prevention, particularly since for some viruses large proportions of transmissions occur soon after infection, regardless of the risk group (Magiorkinis et al., 2013, Brenner et al., 2007, Powers et al., 2011).

Further, molecular sequences have been increasingly used to reconstruct population dynamics in time. The term phylodynamics has been used to describe combination of methods that are based on epidemiologic and phylogenetic techniques for this purpose (Grenfell et al., 2004). Most of the time the phylodynamics approach is used for rapidly evolving pathogens (usually RNA viruses), as these tend to measurably evolve within the host on a similar time scale as they spread between hosts (Kuhnert et al., 2011, Magiorkinis et al., 2013). The phylodynamics methods rely on the hypothesis of the molecular clock which posits that the evolutionary rate of nucleotide sequences can be described by mathematical models, the simplest form being the strict molecular clock with a constant evolutionary rate (Kimura, 1968). Phylodynamics has been extensively used to reconstruct the transmission dynamics of multiple viruses in deeper or more recent time, globally or within specific regions (Magiorkinis et al., 2013, Alizon et al., 2014, Yebra et al., 2015, Zehender et al., 2015).

To study spatial viral disease distribution, phylogeography superimposes geographical information about the molecular sequences on the phylogenetic tree to provide inferences about the spread of the organisms that we are interested in. Phylogeographic methods can be used not only to describe how infectious diseases spread over particular territories, but also to hypothesize what factors (political, socio-economical, and/or ecological) initiated these particular dissemination trends. These methods have been applied within countries and globally to study viruses such as HCV (Pybus et al., 2007, Magiorkinis et al., 2009), HIV (Paraskevis et al., 2009, Angelis et al., 2015, Faria et al., 2014), and Influenza A (Pollett et al., 2015, Alkhamis et al., 2015).

Finally, epidemiological and genetic data can be combined to reconstruct most probable transmission pathways on a community level. For example, the probabilities of the spread of infection between farms during an avian flu outbreak in Netherlands were estimated by taking weighted averages over the set of possible transmission trees (Ypma et al., 2012). The authors concluded that their method provides a more accurate estimate of the transmission pathway than methods based on solely genetic or epidemiological data.

## 2 Epidemics on the micro scale: reconstructing the details of transmission networks

### 2.1 Can we use molecular sequences to infer transmission pathways during infectious disease outbreaks?

Phylogenetic trees reconstructed from genetic sequences contain valuable information about the evolutionary history of the viral strain that can be used to infer possible scenarios of viral infection spread during infectious disease outbreaks. This information is very valuable as it could help to make a prognosis about the further spread of the disease as well as develop control measures in similar epidemiological settings. On a community level a depiction of transmission networks can be estimated by means of phylogeography (Famulare and Hu, 2015). To resolve transmission pathways on an individual level epidemiological contact tracing data are usually used during infectious disease outbreaks. Compared to the contact tracing data that heavily rely on the quality of provided by respondents information and are often subject to self-report bias, genetic data has the advantage of containing unbiased biological information. However, sequencing viral strains quickly as an outbreak develops is challenging for multiple reasons, including unspecific/absence of disease symptoms and/or timely sequencing of the viral strains. Thus, molecular data have been mostly used in a retrospective manner to investigate such infectious disease outbreaks as a nosocomial HCV outbreak in Italy (Spada et al., 2004) and Spain (Gonzalez-Candelas et al., 2013), or on a larger scale for influenza (Jombart et al., 2011). Given that now molecular data can be produced faster and at a lower cost than previously, their use in real-life outbreak investigations becomes more attractive and feasible for some diseases. This has a special promise since portable sequencing technologies such as MinION become available and allow pathogen sequencing in the field (Laver et al., 2015). Recently, genomic data were used in Ebola outbreak investigations to prove that the virus that seeded the outbreak in Guinea in 2014 emerged from Zaire ebolavirus lineage (Dudas and Rambaut, 2014). Similarly, molecular epidemiology methods helped to describe how HIV spread in a community of PWID in several recent outbreaks, including those in Athens, Greece, and Bucharest, Romania (Paraskevis et al., 2015), and in Indiana, USA (Galang et al., 2015; personal communication).

### 2.2 Can we use phylogenetic trees to infer directionality and timing of transmission events?

**Fig. 1:**
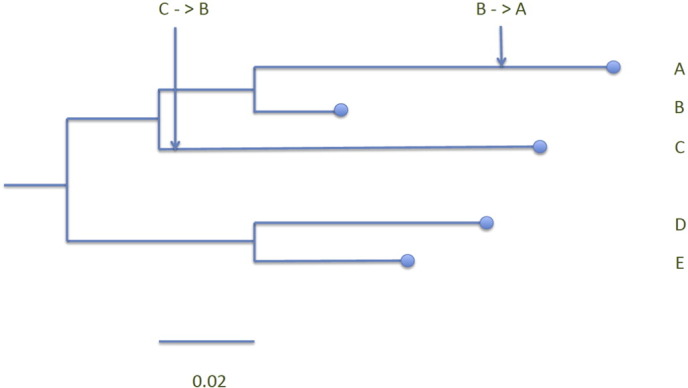
The inferred rooted phylogenetic tree (the root defined using a reference strain not shown on the figure) from a hypothetical known transmission chain. The branch nodes correspond to the coalescent events of the different viral lineages; the arrows show the hypothesized time points when the transmissions happened. Even though it might seem that there was a short time between C ≥ B and B ≥ A transmissions (short genetic distance between the branching and the points of transmission showed by the arrows), in reality it might have been years in between the two events.

Unfortunately, reconstructing phylogenetic trees does not allow us to answer the burning question of “Who infected whom?” (i.e. define the direction of the infection), but only informs us if the two sequences evolved from the same genetic strain. If two sequences are clustered together on a phylogenetic tree, we can say that they have an ancestor in common. Further assigning sampling dates to sequences and implementing molecular clock analyses provides us with an estimate of the timing of the putative transmission events (Leigh Brown et al., 2011). However, these estimates are a subject to bias, because there is a discrepancy between the timing of phylogenetic tree branching that refers to the moment when a viral strain evolved into two and the timing of in-between hosts transmission events. The strain that is transmitted might have evolved within the virus-donor long before the transmission event happened (Fig. 1) (Ypma et al., 2013, Romero-Severson et al., 2014), and, thus, the branching on the tree can overestimate the time of transmission events. This issue can partially be resolved if we can estimate the time of transmission events otherwise. For example, if we sample multiple quasispecies' sequences from each patient (e.g. with NGS, single genome amplification, or cloning), then by running a molecular clock analysis we can estimate the time to most recent common ancestor (TMRCA) of sequences within a patient. This TMRCA can be then superimposed on the phylogenetic tree constructed from sequences obtained from multiple patients.

### 2.3 In which epidemiological settings are phylogenetic trees more useful for transmission pathways reconstruction?

Inferring transmission networks from phylogenetic trees can be problematic for many epidemiological settings. For example, in densely sampled outbreaks of infectious agents phylogenetic trees are likely to have low confidence support. This is especially true when the timescale of the transmission events is comparably fast relative to the evolution of the pathogens, which makes it difficult to infer transmission events based on these phylogenetic trees. Recently, a Bayesian model that takes into account within-host genetic diversity attempted to resolve this issue (Didelot et al., 2014). Even though transmission pathways inferred from genetic data were more ambiguous than those inferred from detailed epidemiological data, the model could still reconstruct some parts of the transmission network, including correctly defining the source of infection in a hypothetical population. An alternative Bayesian model, which also accounts for the within-host virus evolution, but takes into account the non-random host population structure of the epidemic (instead of assuming population panmixis), was suggested for similar settings where dense sampling is available (Hall et al., 2015). It suggests that in a densely sampled outbreak, a well-resolved phylogenetic tree contains the transmission pathway and by sampling different sub-sets of the tree and calculating its posterior probability it is possible to reconstruct a reliable transmission network. The limitation of the later approach is that all of the cases have to be sampled, which is unrealistic for some viral infections, particularly those where a large proportion of cases are unlikely to be diagnosed (like influenza, when many people will be never referred to a hospital) or those that can be asymptomatic for a long time, such as HIV and HCV.

## 3 Social network approach in infectious disease epidemiology

Many pathogens including viruses like HIV and HCV spread non-randomly through networks of closely connected people who engage together in injecting or sexual practices. Consequently, social network analysis has been extensively used as an approach in viral infectious disease epidemiology to recruit participants, monitor and predict behavior patterns, and model further and past disease spread. Initially, the network approach was used a lot to recruit participants into surveys and behavioural studies. Since the mid 1980s epidemiologists faced the problem of obtaining a probability sample of vulnerable to HIV groups, such as those of PWID or men who have sex with men (MSM). Random sampling assumes that every individual and every possible sub-sample within a population has an equal probability to be sampled, which is impossible to define for PWID or MSM, because we don't have a full list of individuals that belong to these groups. Since risky sexual and injecting behaviors are also often stigmatized, contacting PWID or MSM was more difficult than representatives of a general population. Researchers started designing studies based on “snowball” sampling to recruit and study these hard to reach populations (Morris, 2004). One popular sampling strategy is respondent driven sampling, which allows respondents to recruit their peers, but also lets researchers adjust the obtained results to generalize to the whole population (Heckathorn, 1997).

Researchers have integrated social network methods into classical infectious disease epidemiology to study risk factors that enable viral infectious diseases to spread not only at an individual, but at the network level. One of the first network studies, where MSM in California were asked about their sexual partners, found clusters of MSM with AIDS diagnosis who shared sexual partners (Auerbach et al., 1984). This study was of a great importance as it presented epidemiological evidence that AIDS is caused by an infectious agent. Later early network studies on HIV were conducted among drug users in New York (Neaigus et al., 1994, Friedman et al., 1997), female sex workers and PWID in Colorado (Rothenberg et al., 1998), and MSM in California. These studies discovered that the risk to acquire HIV is not only associated with individuals' behaviors; the network position and the behaviors of peers (sexual/injecting partners) play an important role as well (Christley et al., 2005). The network structure might facilitate or limit the spread of viral infections (as well as safe behavior messages) within groups. As a consequence, the social network approach became an important epidemiological tool in the prevention and treatment of viral infectious diseases (Latkin et al., 2013).

Data from real-life social network studies are widely used in mathematical modeling to accurately describe epidemic spread and help to define aims for prevention efforts. The important role of an underlying non-panmictic population structure in epidemic prognosis has been shown for HIV outbreaks a long time ago (Gupta et al., 1989). Populations with assortative mixing of individuals are more likely to experience a rapid epidemic growth early on, while outbreaks in populations with disassortative mixing are more likely to grow into larger epidemics. Later the so-called “firewall” effect was introduced which in theory can be observed when the HIV long-term infected individuals “protect” susceptible individuals from getting in contact with highly infectious acutely infected individuals, inducing saturation at a lower prevalence than the one predicted by a panmictic model (Friedman et al., 2000, Khan et al., 2013, Dombrowski et al., 2013). Improvements in computational capacities have facilitated such advanced epidemiological modeling that takes more complicated population network structures into account (Danon et al., 2011).

## 4 Integrating social and molecular data: the potential of the socio-molecular approach

Similarly to phylogenetic trees, social network data from people who share some risky behaviors include information about possible transmission routes. However, using only network information to resolve transmission pathways is not straightforward: firstly, self-reported behavioural data can contain biased information (especially with regard to socially undesirable/illegal activities); secondly, network studies can almost never recruit all the network members, resulting in incomplete network information. Finally, behavioural data do not necessarily provide evidence about the transmission history of an infectious agent, rather contain information about the shared risks, which might not have led to an infection. For example, sharing syringes/injecting material or practicing unprotected sex with multiple partners can tell about the risks that an individual has undertaken, but cannot be conclusive about the putative source or the date of viral transmission(s). Consequently, combining genetic and social network data into a complex socio-molecular approach might improve the way we infer transmission pathways and reduce the limitations of each other.

*How can the two kinds of data contribute to analysing each of them*? First, recognizing the network structure of a population can help advance the phylodynamics methodology itself. Many phylodynamics methods are based on the coalescence model that falsely assumes that host populations are panmictic—that is, that every host has an equal probability to contact and transmit the viral infection agent to another individual, which is not the case in real life. On the contrary, the network population structure of hosts implies preferential mixing by the number of partners (i.e., “highly active people tend to have highly active partners”) and often within social groups (like race/ethnicities) or across sexual groups (men with women more than men with men or women with women) (Goodreau, 2006). Consequently, it leads to heterogeneity in the number of secondary infections, i.e. some people transmit to more people than others, giving birth to more new infections (Goodreau, 2006). Models have been developed to estimate the effect of this heterogeneity on the phylogenies. Some researchers have found that the distribution of the number of secondary infections in a population have an effect on reconstructed pathogen phylogenies (Robinson et al., 2013). Heterogeneity in the number of secondary infections results in phylogenies with more clusters of a smaller size and shorter mean branch lengths compared to phylogenies reconstructed from a population with homogeneity in the number of secondary infections. Introducing this kind of more complex phylodynamics models that account for the network structure of a population may allow more accurate estimates of transmission chains.

**Fig. 3:**
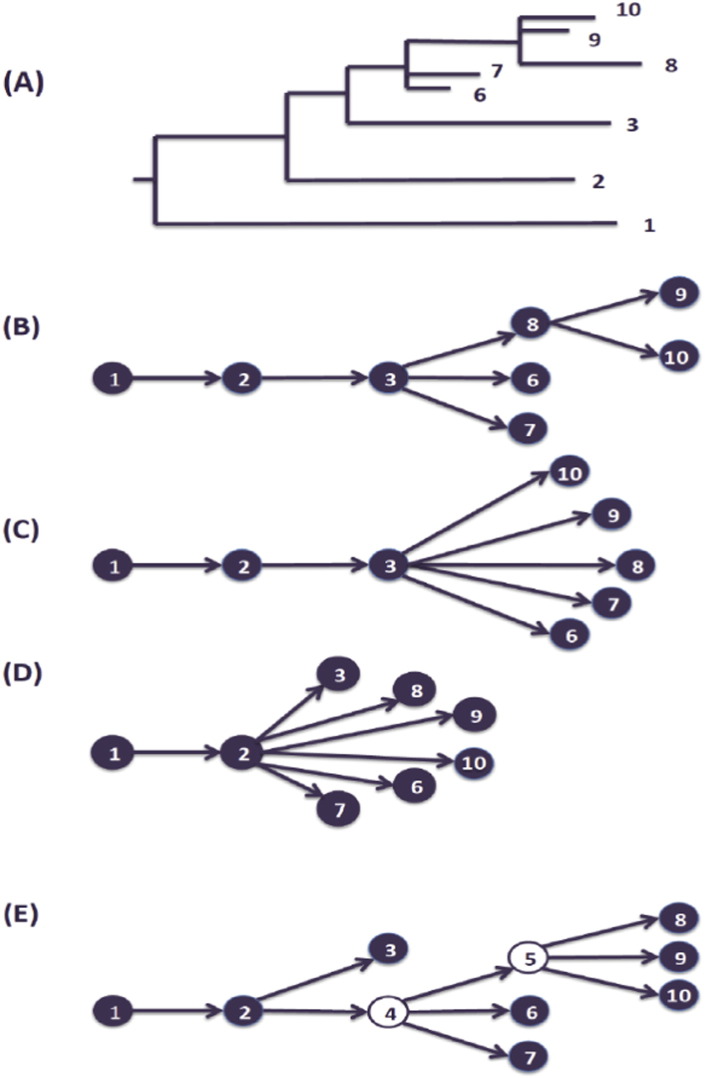
(A) A phylogenetic tree, inferred from sequences obtained from a set of individuals that form a network sharing a risky behavior; (B), (C), and (D) Some of the transmission pathways that could be inferred from this phylogenetic tree. If participants 2, 6, and 7 provide information about unsampled/cleared individuals number 4 and 5, the transmission pathway can be better resolved to describe the actual transmission pathway (E).

In practice, social network information can sometimes be applied to resolving ambiguous or equally plausible transmission pathways reconstructed from phylogenetic trees if genetic and social data come from the same individuals. One of the main differences between the social and the genetic data is that the social network data may theoretically include an overall contact network (as in Fig. 2.1), including non-infected individuals, even individuals who were never recruited (but reported to be part of the network by other members). Knowing additional information about other members of the network, whose viral genetic information was not sampled for some reason, can help to choose one out of multiple plausible transmission pathways inferred from a phylogenetic tree (Fig. 3). For example, for many infectious agents, spontaneous clearance is part of the natural history of the disease. This makes it difficult to rely on phylogenies in an attempt to reconstruct transmission pathways: there are individuals who might have transmitted the pathogen in the past, but at the time of sampling have successfully cleared the infection (Brewer et al., 2006). In this case, viral strains cannot be retrieved for sequencing and phylogenetic analyses, but the epidemiological data from disease-free network members can include self-reported information about their previous disease status. Even more, an antibody positive test might indicate that a person used to be infected, while the type (e.g. IgM or IgG) and specificity (e.g. avidity test) of the antibodies, might provide us with information about the recent or non-recent nature of a transmission. Thus, connections that lead to transmissions might not be captured in the phylogenetic tree, but social network information and epidemiological data might help to fill in the gaps on a hypothetical transmission pathway.

Social network information in theory can bring many insights onto how to interpret the phylogenetic trees, although the methods to do this have yet to be developed. Supposedly, knowing the network position of sampled individuals can help to estimate how reliable are the transmission pathways estimated from the phylogenetic trees. As previously said, in most settings, it is too optimistic to think that all of the network members can be recruited (or specimens from all the infected collected, either), and incomplete sampling can bias the analyses. Luckily, in social network studies respondents are usually asked about other members of the network. Theoretically, this information about other members of the network can help to place additional nodes at the transmission networks estimated from phylogenetic trees helping to resolve ambiguous transmission patterns. Further, hypothetically, sampling individuals who have more central position in a network allows estimating phylogenetic trees that are more likely to contain a real transmission pathway (Fig. 2.1, 2.2). At the same time, sampling individuals with less central position in a network might result in multiple phylogenetic trees, thus, making an attempt to reconstruct the true path of the virus spread more challenging.

Also, social network data and phylogenetic trees usually refer to different time points and using the estimated timing of the transmission events can provide insight on dynamic changes in the structure of a transmission network. Contact data collected in social network studies describe the most recent connections among individuals. Most social network studies have only addressed a short period of time, because relationships among individuals change rapidly and, additionally, recall bias is higher with respect to older connections (Bell et al., 2007). Phylogenetic trees on the contrary can infer past events that happened when the viruses evolved within infected individuals. For chronic viral infections these events might have occurred many years ago. Thus, combining the two data sources can provide complementary insights about changes in social structure of the population of hosts and, consequently, transmission networks.

## 5 Current applications that combine multiple data sources

Trying to reconstruct possible transmission chains from phylogenetic data, some researchers have relied on bootstrapping as a way to identify probable ties (Lewis et al., 2008). Leigh Brown et al. used molecular clock analysis of HIV in combination with epidemiologic data obtained from one social network study. The authors used genetic sequences (one per patient) as nodes and links were made if the most recent common ancestor of two nodes went back in time not more than a defined period of time (e.g. less than 3 years) (Leigh Brown et al., 2011). They then compared the distribution of the average number of sexual partners in MSM that they got from the networks constructed from phylogenies to the numbers obtained in surveys. They obtained similar highly right-skewed distributions of the number of links from epidemiological and genetic data.

Several research groups have used additional epidemiological information (patient's risk group) assigned to the viral sequences to study whether transmissions happen within contact networks of particular sub-populations; they found that population mixing in the groups they studied was assortative and there was little bridging between the risk groups (Yebra et al., 2015, de Bruijne et al., 2009, Lunar et al., 2015). Other researchers found, on contrary, that HIV epidemics in one group can be seeded by introductions from other risk groups within the same country. For example, in some European countries, HIV epidemics among heterosexuals were seeded and sustained by transmissions from PWID (Kouyos et al., 2010, Graw et al., 2012). Kouyos et al. were able to identify sexual transmission of HCV in cases of HIV-infected MSM and heterosexuals by combining genetic information and clinical and epidemiological information (patients' risk group and HCV serostatus), improving our knowledge about transmission routes and epidemiology of HIV-HCV co-infection (Kouyos et al., 2014). Revealing such patterns of transmission between groups can assist in designing intervention strategies.

Some attempts to apply the socio-molecular approach to study viral transmissions have compared social and viral genetic distances between individuals. Several studies used HCV spread in PWID as a model system. Such, multiple data sources were combined to look at the association between genetic relatedness of the HCV sequences (within genotype groups) and social distances between PWID in two studies from Melbourne, Australia (Aitken et al., 2004, Sacks-Davis et al., 2012). The authors of both studies found a weak correlation between social geodesic distance (the smallest number of injecting partnerships connecting two nodes) and HCV genetic distance. They have explained this by incomplete sampling, potentially biased self-reported data, long carriage of HCV, and, most importantly, the long injecting history of participants (over 10 years) and long duration of injecting partnerships. The authors suggested that cohorts of recent drug injectors or recently infected individuals might be more appropriate to search for an association between social and genetic distances. This was partly addressed by another study of HCV in PWID in Seattle, Washington. The authors of this research looked at the relationship between social and genetic distances between recently HCV-infected participants (anti-HCV-negative < 8 months ago). Initially, the authors found an association between social and genetic distances. However, this seemed to be due to one influential point (one confirmed transmission pair with reported needle sharing and shared common ancestor). When this pair was removed from the phylogenetic tree the association was lost (Brewer et al., 2006). This suggests that even when social and genetic data are collected at the same period of time, there may sometimes be low or no correlation between the two measures.

Looking for similarities between social and genetic patterns, researchers have compared phylogenetic clustering of HCV and social network structures of PWID. The same study from Melbourne that found a weak association between the two distance measures when only individuals and their ties were considered found an association between a social partnership (self-reported injecting in the same place and at the same time) and being in the same phylogenetic cluster (Sacks-Davis et al., 2012). This suggests that including information about venues where people engage in risk behaviors can help to find associations between genetic and social data. Another group of researchers searched for the association between phylogenetic clustering of both HIV and HCV and social partnership (defined as the distance on their recruitment chain) within injecting networks of PWID in Ottawa, Ontario, Canada (Pilon et al., 2011). An interesting finding was that participants co-infected with both infections were not always concordantly clustered within the phylogenies of HIV and HCV. Thus, participants who were clustered on an HIV phylogenetic tree were clustered on an HCV phylogenetic tree only in 50% of the cases. Again, the authors reported that there was a lack of support of social network information from the phylogenetic trees: if two HIV-positive people recruited each other, their specimens never shared a common viral ancestor (for HCV 10% of phylogenetic clustering resulted from recruitment). However, recruitment chain proximity can be a poor proxy for a social partnership/distance.

Although there is no “conventional” way to combine genetic and social network data, several approaches have been suggested up to now. These include network simulations, comparisons between real-life data, informing network data based on the social information and vice versa. Goodreau modelled several social network patterns, consequently simulating HIV spread in those networks and then the viral mutations among infected hosts (Goodreau, 2006). He used the coalescence approach to estimate *N*_e_ (effective population size which equals the size of an idealized population that shows the same genetic drift as the studied population) under different underlying population structures. He concluded that these estimates for some population structures are similar to those obtained under the assumption of population panmixis. Still, some social network patterns, like those resembling venue-centered networks of female sexual workers, surprisingly produced much higher estimates of *N*_*e*_ than the actual population size. Additional research is needed to define if for some risk groups phylodynamic estimates are less reasonable than for the others.

The socio-molecular approach in epidemiology is at its starting point. Many researchers try to find the best way to use both social and molecular data to improve different aspects of infectious disease epidemiology. There are several issues that prevent from wider use of network data. First, the cost of collecting network data is high; secondly, many infectious diseases (e.g. HIV), are associated with stigma that demotivates participants to participate in the studies and/or refer their partners; finally, network surveys often include sensitive questions about sexual and injecting partners, which renders these studies ethically challenging and potentially raises safety issues for the field researchers. However, some main obstacles of previous years, such as computational complexity of the network analysis and expensive and time-consuming sequencing are greatly relaxed, suggesting that a wider use of social and molecular approaches is feasible, and at the same time raising interesting questions. How to relate social data to molecular? What questions can be asked with the two sources that never would have occurred to us with only one source? How reliable are transmission pathways estimated from both sources? Will the combination of the two improve our understanding of how transmissions happen or will they contradict to each other? Can combining the two methods assist in case finding or other interventions? Further research on how the socio-molecular approach can validate data obtained from one of the sources, overcoming limitations, or relaxing assumptions of epidemiological methods will help answering these questions.

## Figures and Tables

**Fig. 2:**
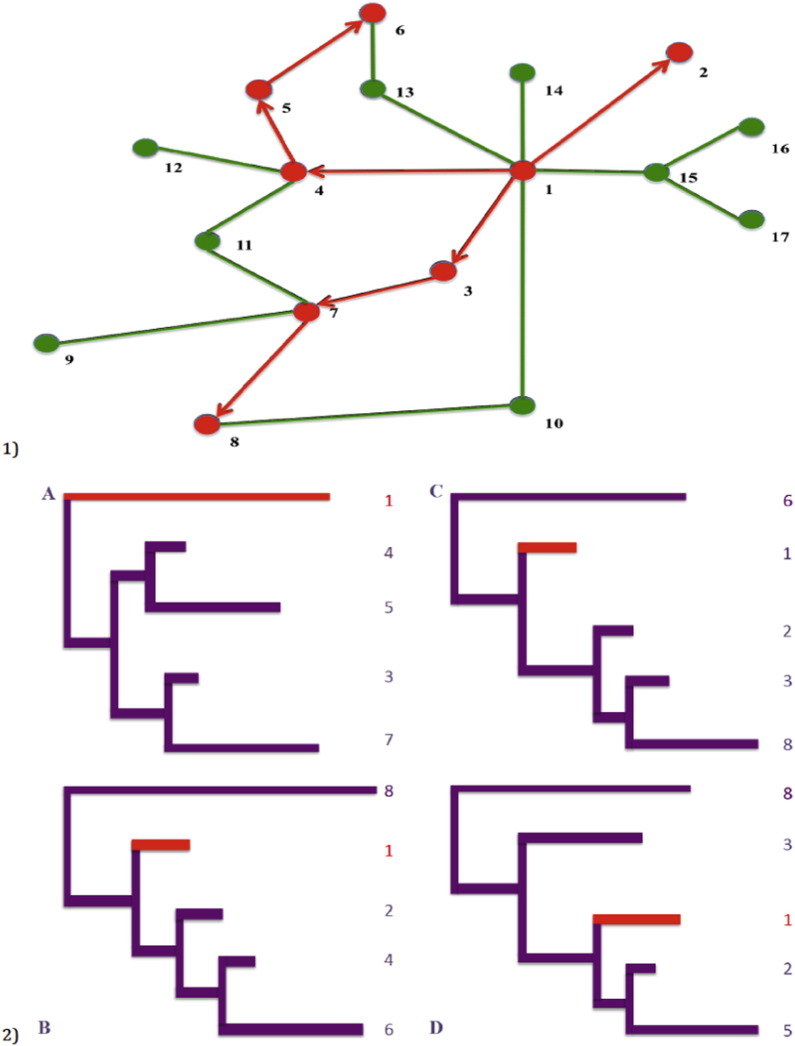
1 A hypothetical contact network of PWID with known transmission pathway. Red circles – HIV-infected individuals, green circles – HIV-free individuals. Red arrows indicate the direction of infection (who infected whom), green lines indicate an injecting partnership that did not lead to an HIV transmission. 2 Phylogenetic trees reconstructed from a subset of a network of PWID. Panel A represents the phylogenetic tree based on the samples collected from the individuals 1, 3, 4, 5, 7 (who have higher degree of centrality). Panels B, C, and D represent phylogenetic trees reconstructed from the samples collected from individuals who have lower degree of centrality.
